# Good practices in normal childbirth: reliability analysis of an instrument by Cronbach’s Alpha^1^

**DOI:** 10.1590/1518-8345.2234.3000

**Published:** 2018-05-17

**Authors:** Leila Bernarda Donato Gottems, Elisabete Mesquita Peres De Carvalho, Dirce Guilhem, Maria Raquel Gomes Maia Pires

**Affiliations:** 2 PhD. Professor. Escola Superior de Ciencias da Saude, Fundação de Ensino e Pesquisa em Ciencias da Saude, Brasilia, DF, Brazil.; 3 MSc. Doctor degree student. Post Graduate Program in Health Science, Escola Superior de Ciências da Saúde e Universidade de Brasília, Brasília, DF, Brazil. Doctor Degree Interinstitutional in Health Sciences. RN. Superintendencia da Região de Saude Norte, Secretaria de Estado da Saude do DF, Brasilia, DF, Brazil.; 4 PhD. Professor. Departamento de Enfermagem, Universidade de Brasilia, Brasilia, DF, Brazil. Bolsista de Produtividade em Pesquisa do CNPq - Nível 1-D.; 5 PhD. Adjunct Professor. Departamento de Enfermagem, Universidade de Brasilia, Brasilia, DF, Brazil.

**Keywords:** Validation Studies, Data Accuracy, Statistical Analysis, Perinatal Care, Health Evaluation, Humanizing Delivery

## Abstract

**Objectives:**

to analyze the internal consistency of the evaluation instrument of the adherence to the good practices of childbirth and birth care in the professionals, through Cronbach’s Alpha Coefficient for each of the dimensions and for the total instrument.

**Method:**

this is a descriptive and cross-sectional study performed in obstetric centers of eleven public hospitals in the Federal District, with a questionnaire applied to 261 professionals who worked in the delivery care.

**Results:**

The study was attended by 261 professionals, 42.5% (111) nurses and 57.5% (150) physicians. The reliability evaluation of the instrument by the Cronbach Alfa resulted in 0.53, 0.78 and 0.76 for dimensions 1, 2 and 3, after debugging that resulted in the exclusion of 11 items.

**Conclusions:**

the instrument obtained Cronbach’s alpha of 0.80. There is a need for improvement in the items of dimension 1 that refer to attitudes, knowledge, and practices of the organization of the network of care to gestation, childbirth, and birth. However, it can be applied in the way it is used to evaluate practices based on scientific evidence of childbirth care.

## Introduction

The incorporation of good practices in childbirth and birth care is one of the main strategies for changing the obstetric model, reducing maternal and infant morbidity and mortality, and the access to quality services, as recommended by the World Health Organization (WHO), repeated by the health policies in Brazil^1^ ^-^ ^7^.

In the Brazilian context, it is worth mentioning the policy called “Rede Cegonha”, published by the Administrative Rule number 1459, of June 24, 2011. It is proposed the organization of services in healthcare networks with emphasis on the articulation between the prenatal care and places of delivery, strengthening the adoption of evidence-based practices by health professionals, presence of the partner, health education for parturients, family members and companions, and continuing education of professionals, to enhance changes in care directed to women and to the child^1^ ^,^ ^4^ ^-^ ^5^. Also, the national guidelines for assistance to normal childbirth, proposed by the National Council for the Incorporation of Technologies in the Unified Health System, published in 2016, guide professionals in their daily activities, with a systematic and synthesized evaluation of available scientific information, to be able to make day-to-day decisions.

However, the production of changes in delivery and birth care remains a complex challenge for managers, researchers and social movements^1^ ^-^ ^7^. Within health services, the reorientation of practices can be enhanced by evaluation for the management of health services^8^ ^).^ It is a technical-administrative and political process of judging the value or merit of something, to subsidize managers in making everyday decisions, based on the use of research methods and techniques in their design, formulation, and implementation^(8- 9)^. In particular, evaluative research is needed to address the middle or operative level of management, where actions occur from macropolitical decisions and manifested in the care model, work processes, resource drawing up the programs^9^.

The scientific literature has shown gaps in the understanding of the potentialities and limitations in the work of professionals who work in childbirth care, with low adherence to good obstetrical practices^2^ ^-^ ^3^ ^,^ ^7^. Adherence is a dynamic, multifactorial, and behavioral process that results from a set of determinants that depend on subjective factors such as personality traits, cognitive and intellectual level, beliefs, and social context of which the person is a part. The terms adherence and compliance have been used to designate the degree of coincidence between the behaviors of the individual (patient or client) and the therapeutic recommendations of the health professional^10^. In this study, adherence was adopted as the coincidence between the knowledge, attitudes, and practices of the professionals regarding the technical recommendations and scientific evidence on the childbirth care^11^.

The instrument of measures analyzed in this article is focused on the context of the health services, the multiple knowledge of the professionals, the values, beliefs, and ideas that intermediate the relationship established with the parturients^10^ ^-^ ^11^. It was constructed based on the practices proposed by the “Rede Cegonha” guidelines, treated as constructs to be measured (latent variable) through indirect observation of their manifestations in the daily work process in health, in the network of childbirth and birth care^12^. These manifestations were transformed into items (operational concepts) that constitute attitudes, knowledge, practices, behaviors, and opinions about the health and physical and psychological well-being of women^11^ ^-^ ^13^.

The research data were used to test the validity (ability to measure what is proposed in a certain phenomenon) and the reliability (ability to present measures faithful to reality) of the instrument^14^ ^-^ ^15^. Thus, this article had the objective of analyzing the internal consistency of the evaluation instrument of the adherence of the professionals to the good practices of childbirth and birth care, using the Cronbach Alpha Coefficient for each one of the dimensions and for the total instrument.

## Method

A descriptive and cross-sectional study with a quantitative approach was carried out in eleven obstetric centers of public hospitals of the State Department of Health of the Federal District. Data were collected from January to March 2015. The instrument was applied to physicians, nurses, and residents (medicine and nursing) who worked in the direct care of childbirth. The sample was composed of 261 health professionals, stratified by hospital, according to the number of professionals working in each hospital. This sample was calculated based on the rule of thumb, with at least 5 respondents per item of the instrument, equivalent to at least 250 respondents considering that the instrument has 50 items^14^ ^-^ ^16^.

The original instrument is divided into three dimensions: Organization of the Network for Care to the Gestation, Childbirth, and Birth (items 1 to 12), Practices Based on Scientific Evidence (items 13 to 35) and Work Processes (items 36 to 50). It also contains questions about the socioeconomic, demographic and professional profile^11^. The items of the instrument referring to the professional practices follow the scale of five Likert points, transformed into values from 0 to 100, so ordered and scored: disagree completely (1=0 point), disagree partially (2=25 points) , does not know/does not apply (3=50 points), partially agrees (4=75 points), totally agrees (5=100 points).

The profile data were analyzed using descriptive statistics. Cronbach’s alpha was calculated to evaluate the internal consistency of the instrument. It is a coefficient that measures the correlation between the answers in a questionnaire through the analysis of the profile of the answers given by the respondents, whose values vary from 0 to 1^14^ ^-^ ^17^. The closer to 1, the greater the reliability of the indicators. A generally accepted lower limit is 0.7, although it drops to 0.6 in exploratory research. The Cronbach Alpha Reliability classification occurs as follows: Very low (α ≤ 0.30); Low (0.30 <α ≤ 0.60); Moderate (0.60 <α ≤ 0.75); High (0.75 <α ≤ 0.90) and Very high (α> 0.90)^14^ ^-^ ^17^.

Reliability (intensity of correlation between items) was further tested by eliminating items from the questionnaire in the debugging process. If with the elimination of an item, the Coefficient increased, it was assumed that this item was not highly correlated with the other items of the scale and it could be eliminated from the instrument. If the Coefficient decreased, it was assumed that this item was highly correlated with the other items of the instrument. Alfa Cronbach was calculated for the instrument as a whole and for each dimension^14^ ^-^ ^17^.

The project was approved by the Research Ethics Committee of the State Department of Health of the Federal District, under the number CAAE 01918712.6.0000.5553. The study was funded by the Research Support Foundation of the Federal District (Process nº 193.000.175-2013).

## Results

There were 42.5% (111) of nurses and 57.5% (150) of physicians among the 261 professionals who participated in the study. The mean age was 35 years old for nurses (±9.49) and 39.47 (±10.17) for physicians. The time of operation in the delivery room was on average 5 years for nurses (±5.41) and 12 years for physicians and the mean time of training was 10.37±8.00 for nurses and 14.4410.48 for physicians. In the distribution by gender, 92% were female in nursing and 68% were female in medicine. The weekly workload averaged was 44 hours for nurses and 45 hours for physicians.

In the analysis of the instrument´s internal consistency, the Cronbach Alpha result of Dimension 1 was 0.49 with the original items of the instrument (Table 1). Correlation values ranged from 0.44 to 0.38, considered moderate. Variables 1 and 7 presented the lowest correlations. After its exclusion, Alfa Cronbach increased to 0.51 and to 0.53 when also excluded item 8.

**Table 1:** Mean, standard deviation, item-total correlation coefficient, and α-Cronbach if the item is excluded from Dimension 1- Organization of the Network for Care to Gestation, Childbirth, and Birth. Brasilia, DF, Brazil, 2015

Variable	M*	SD^†^	Correlation item-total	α Cronbach if excluded
V1 - I do not know the area of coverage of this service of delivery and birth.	82.85	30.39	0.04	0.51
V2 - I got a bed in another care unit when there was no vacancy in this service.	64.08	35.97	0.16	0.48
V3 - I get pregnant to know the place of birth, routinely.	52.11	38.67	0.32	0.43
V4 - Educational activities are carried out with the pregnant women and partners to favor the attachment to maternity when the previous visit.	54.69	38.47	0.38	0.41
V5 - We usually treat more parturients than the number of beds.	12.36	25.25	0.25	0.46
V6 - The staff is insufficient for the number of parturients attended daily.	17.24	30.38	0.28	0.45
V7 - We receive pregnant women without the individual birth plan done during prenatal care.	13.79	27.23	0.05	0.5
V8 - In general, we received the women without the results of the prenatal risk screening tests.	32.18	32.87	0.18	0.47
V9 - We can easily contact the prenatal team of primary care and/or the high-risk clinic when needed.	31.70	33.73	0.12	0.49
V10 - I participate in meetings with prenatal teams to discuss improvements in gestation, delivery and birth care.	26.92	35.51	0.13	0.49
V11 - When we need support, diagnosis and therapeutic procedures that are not available, we have difficulties in getting other services.	21.36	30.48	0.19	0.47
V12 - Access to information made by the other health units is facilitated by the electronic medical record.	66.38	33.86	0.14	0.48

*M=Mean; †SD=Standard Deviation

In Dimension 2, a Cronbach’s alpha of 0.74 with all items was obtained, without any exclusion, as observed in Table 2. After exclusion of items 33 and 35 that presented negative item-total correlation, the Alpha of the dimension reached 0.78. Cronbach’s alpha values were moderate in all variables, without the exclusions.

**Table 2:** Mean, standard deviation, Total-Item correlation coefficient, and Cronbach’s α if the item is excluded from Dimension 2 - Practices Based on Scientific Evidence. Brasilia, DF, Brazil, 2015

Variable	M*	SD^†^	Correlation item-total	α Cronbach if excluded
V13 - Normal delivery is performed in PPP^‡^ beds at this service.	84.96	29.52	0.19	0.74
V14 - I restrict the fluid intake and food intake of the parturient during the TP^§^, routinely.	64.56	35.47	0.32	0.73
V15 - I use curtains and/or screens to preserve the parturient’s privacy in the prenatal group.	78.07	33.97	0.3	0.73
V16 - I encourage the presence of free choice partner of the parturient.	78.26	33.17	0.32	0.73
V17 - I recognize that the partner hinders the woman´s care.	65.90	35.64	0.34	0.73
V18 - The partner is rarely informed about the condition of the parturient.	75.29	32.78	0.21	0.74
V19 - Guidance on ways of relaxation for pain relief during TP^§^ and childbirth.	85.06	24.26	0.39	0.73
V20 - I stimulate the ambulation of pregnant women during TP^§^.	93.39	16.02	0.36	0.73
V21 - I use non-pharmacological methods for pain relief, such as massage and relaxation techniques.	67.82	33.95	0.42	0.72
V22 - I encourage the freedom of the woman’s position during the TP^§^ and childbirth.	81.90	26.41	0.45	0.72
V23 - In the active phase of the TP^§^, I undergo heart rate auscultation every 30 min, routinely.	70.40	30.90	0.36	0.73
V24 - I use a deliverygraph to follow the TP^§^.	50.00	39.65	0.44	0.72
V25 - I offer information to the parturient on the TP^§^.	90.13	21.28	0.36	0.73
V26 - I promote skin-to-skin contact between mother and child within the first half hour after childbirth.	91.19	19.21	0.32	0.73
V27 - The enema is routinely done in preparation for childbirth.	96.26	13.97	0.12	0.74
V28 - The trichotomy is performed routinely in this service.	88.98	25.59	0.22	0.74
V29 - Intravenous hydration is used during the TP^§^ and childbirth.	35.92	31.17	0.36	0.73
V30 - Intravenous oxytocin is used to TP^§^.	31.13	26.14	0.37	0.73
V31 - I encourage the parturient to push at the time of the expulsion of the fetus.	19.64	29.33	0.43	0.72
V32 - A routine episiotomy is performed in this service.	54.89	34.43	0.29	0.73
V33 - It is avoided to perform vaginal touches by more than one professional.	56.13	36.50	-0.12	0.77
V34 - I perform Kristeller´s maneuver when necessary.	54.21	38.75	0.43	0.72
V35 - Early amniotomy is rarely performed in this service.	66.57	34.12	-0.03	0.76

*M=Mean; †SD=standard deviation; PPP‡- Pre-childbirth, childbirth and puerperium; TP§ = Childbirth

**Table 3:** Mean, Standard Deviation, Coefficient of Item-Total Correlation, and Alpha Cronbach if the item is excluded from Dimension 3 - Work Processes. Brasilia, DF, Brazil, 2015

Variable	M*	SD^†^	Correlation item-total	α Cronbach if excluded
V36 - I follow the recommendations of the Ministry of Health the childbirth and birth care.	86.21	18.38	0.28	0.61
V37 - Parturients are informed before the interventions that accelerate the TP^‡^.	80.46	28.19	0.49	0.57
V38 - In this service, clinical decisions are shared with the on-call staff.	71.84	33.57	0.63	0.54
V39 - Each professional attends the delivery according to their experience.	26.25	30.32	-0.14	0.66
V40 - I discuss the scientific evidence on childbirth and birth care with my team.	71.07	31.26	0.40	0.58
V41 - I feel out of date with the scientific evidence.	66.86	36.27	0.29	0.60
V42 - Doctors and nurses work in an integrated way in this service.	62.36	33.51	0.42	0.58
V43 - Here normal labor is stimulated by the multi-professional team.	79.12	26.94	0.50	0.57
V44 - I assist low-risk childbirth similar to high-risk childbirth.	58.81	37.65	-0.30	0.70
V45 - In this service, the professionals are trained periodically.	47.99	32.05	0.45	0.57
V46 - Here patients´ satisfaction surveys are carried out.	25.77	28.58	0.37	0.59
V47 - I record information about care at childbirth and birth only in the electronic medical record.	67.53	37.17	0.12	0.63
V48 - I rarely consult the information about prenatal care registered on the pregnant woman’s record.	92.62	21.27	0.11	0.62
V49 - The training offerings for the professionals of this team are rare.	30.27	33.30	0.17	0.62
V50 - Nursing care is restricted to some shifts (scarce).	31.99	35.58	0.23	0.61

*M=Mean; †SD=standard deviation; ‡TP=Childbirth;

In Dimension 3, with all the original items, Cronbach’s Alpha of 0.62 (moderate) was obtained. After the removal of items 39, 41, 44, 47, 48 and 49, whose item-total correlations were low and/or negative, the Alpha value of 0.766 was obtained.

**Figure 1:**
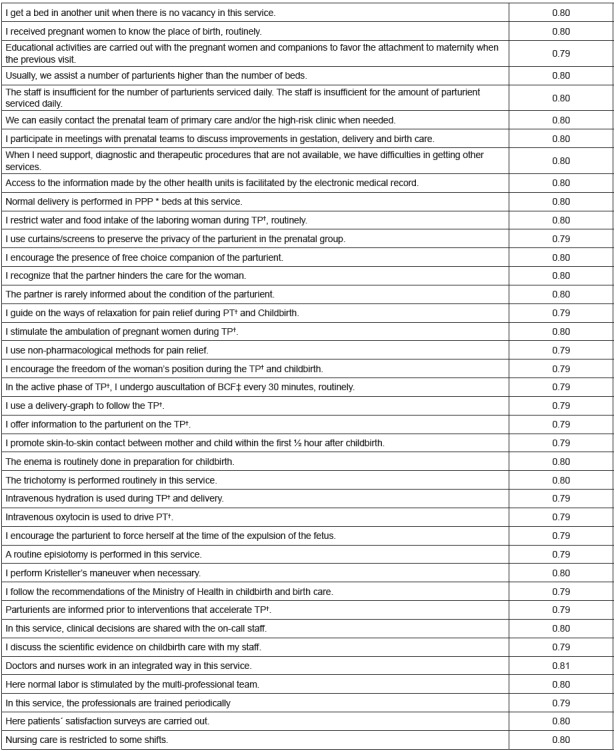
Items recommended for the instrument “adherence of the professionals to the good practices of childbirth and birth care” with the respective α-Cronbach values obtained after the clearance. Brasilia-DF, 2015

The instrument obtained a Cronbach Alpha total of 0.745 with all items. Excluding items, V1 and V7 of Dimension 1, V 33 and 35 of Dimension 2 and V39, V41, V44, V47, V48 and V49 of Dimension 3, a value of 0.80 were obtained. After debugging, the instrument had 39 items, according to Figure 1.

*PPP= Pre-childbirth, childbirth and puerperium; †TP= Labor and Delivery; ‡BCF- Cardiopulmonary;

Cronbach’s alpha was calculated according to gender and professional category. All items had an alpha of 0.75 for both genders. After the 11 items were excluded, 0.80 was obtained for the female population and 0.79 for the male population. In the comparison of Alpha among physicians and nurses, the total number of items was 0.77 for nurses and 0.73 for physicians. After the exclusions, it was 0.82 for nurses and 0.78 for physicians.

## Discussion

In the reliability analysis of the instrument by Cronbach’s Alpha, it was possible to obtain an improved proposal of the questionnaire “adherence to good practices in the normal birth care” (11). Dimension 1 presented a coefficient of 0.53, with low correlation among items, therefore, they had a low reliability. The alpha value was low (0.30 <α ≤ 0.60) little changed with the exclusions. However, very low values (α ≤ 0.30) were not obtained in any of the tests. The purification process was not enough to increase Alpha, demonstrating that the construct requires improvement in how to operate it in items^16^ ^-^ ^17^.

The restructuring of care for pregnant women and the newborn, with a link between prenatal care in primary care and childbirth care in the hospital setting, are the main tools introduced by the “Rede Cegonha”^1^ ^-^ ^4^. However, they still require the incorporation in the management of health services of tools that favor the systemic view of professionals to be consolidated as practices of health professionals, such as action plans, linking maps between units and technologies that favor the exchange of information between health units and professionals^1^ ^-^ ^2^ ^,^ ^11^. This may explain the moderate consistency of this dimension (0.60 <α ≤0.75)^14^ ^-^ ^15^.

In Dimension 2, after clearance, Cronbach’s alpha was high in the total dimension and in all items (high 0.75 <α ≤ 0.90)^14^ ^-^ ^15^. The two excluded items refer to the performance of vaginal touches by more than one professional and to the use of early amniotomy in the services, both reversal items in the instrument and unnecessary obstetric interventions^2^ ^-^ ^7^.

The evaluation of the adherence to practices based on scientific evidence demonstrated in the items of Dimension 2 showed that professionals both perform good practices and still intervene unnecessarily in childbirth. The results are consistent with data from other national studies, which demonstrate that episiotomy is still performed in 56% of vaginal deliveries; that lithotomy delivery occurs in 92% of the women and that 37% of the mothers underwent Kristeller’s maneuver, none of them supported by the best available evidence^4^ ^-^ ^5^. Positive practices such as feeding during labor (26%) and freedom of movement in labor (46%) are also pointed out in the literature. Studies in international settings such as Tehran and Latin American countries, including Brazil, also demonstrate the concomitant adoption of good practices and unnecessary interventions among professionals^18^ ^-^ ^19^.

There is a need for important actions to favor the incorporation of simple recommendations, such as wandering or not maintaining routine venous access^6^ ^-^ ^8^. Devices such as birth plans constructed from basic health units during prenatal care deserve to be taken up to help professionals and women to jointly rebuild the technicality of gestation, childbirth and birth care in favor of a preventive, contemplative and humanistic attention^1^ ^-^ ^7^ ^,^ ^18^ ^-^ ^21^. The incorporation of practices with scientific foundations and respecting childbirth may be very rewarding for health professionals, but implying a re-signification of asymmetric power relationships^18^ ^-^ ^20^. Regarding the abandonment of interventions, it is necessary to follow the path taken by other countries, such as the United States of America^20^, in which the consumer groups and activists, in combination with institutional support, reinforced the need for a caring approach based on evidence, with the aim of contributing to improvement in outcomes, without the iatrogenic damage associated with excessive interventions^1^ ^-^ ^7^ ^,^ ^21^.

The third dimension obtained Alpha of 0.766 after debugging. Excluded items represent relevant but undesirable behaviors among professionals, such as attending delivery according to their experience(39), being out of date with scientific evidence (41), attending low-risk childbirth similar to high-risk delivery (44), registration of information on delivery care and birth only in the electronic medical record (47), rarely consult the information about prenatal care on the pregnant woman’s record (48) and a low offer of training to professionals (49).

It is important to highlight that the work process requires a permanent education in procedures, in clinical protocols, in the construction of information and decision-sharing spaces, and in technological support^1^ ^-^ ^7^. Although clearance has substantially altered the result by showing that the excluded items had low consistency, the researcher’s decision about the relevance or otherwise of the exclusion is necessary^14^ ^-^ ^15^. It is observed that all are negative items that may have influenced the result and refer to extremely relevant aspects of the work process.

The purification process resulted in an instrument with Cronbach’s Alpha total of 0.80, considered as satisfactory for measuring instruments^16^ ^-^ ^18^. It contains 39 items, according to Figure 1. Reducing the number of items, although, without consensus in the literature on ideal size, it may represent advantages for new applications^16^.

It is important to emphasize that this coefficient assists the researcher on the pertinence or not of an item in a given questionnaire, but does not replace the decision on the relevance of the item within the general context of the construct under study^14^ ^-^ ^15^. For this reason, although they did not obtain Alfa Cronbach high, the items of the first dimension were maintained. There is a need for further studies to improve the internal consistency of the instrument for use in the evaluation of knowledge, attitudes, and practices aimed at the organization of integrated systems for care to gestation, childbirth, and birth^16^.

Another result to be observed was the difference of Cronbach’s Alpha between gender and between the professional categories of the study. By the results, women and nurses are more sincere in the answers. This data is relevant since the values of Alfa can be altered by other characteristics of the sample in new applications of the instructor^14^ ^,^ ^16^.

It is important to highlight that the robustness of the results of a study depends on the instrument used that must have internal consistency and quality^14^ ^-^ ^16^. The evaluation of health services still presents a challenge, considering the complexity of the object to be evaluated, the difficulties in systematizing evaluation tools and in obtaining reliable data, documents and information for this purpose^18^ ^-^ ^21^. There are gaps in knowledge and the need for research that develop tools to measure the quality of care and the continuous improvement of facilities, attitudes, behavior and power relationships among health professionals. The application of this instrument can subsidize actions to improve the quality of delivery care^18^ ^-^ ^20^.

## Conclusion

The value of the total Cronbach’s Alpha coefficient of the instrument after the debugging was 0.80 with 39 items. Among the dimensions, this coefficient ranged from 0.53 to 0.76. The first dimension presented a low correlation with the other items, which requires a new application after revision to achieve a higher reliability index. However, as it stands, it can be applied to evaluate the knowledge, attitudes, and practices of professionals who work in childbirth care.
